# Supplementary material for: HIVprotI: an integrated web based platform for prediction and design of HIV proteins inhibitors
Source: J Cheminform. 2018 Mar 9;10:12. doi: 10.1186/s13321-018-0266-y (PMC5845081; doi:10.1186/s13321-018-0266-y)
Supplement: Supplementary file 2 — Additional file 2. Source code of HIVProtI web server. [file 13321_2018_266_MOESM2_ESM.zip › HIVprotI_Source-code/hivprotI/weka-3-6-6/doc/weka/core/FindWithCapabilities.html]

FindWithCapabilities


---


|  |  |  |
| --- | --- | --- |
| **Overview** \| **Package** \| **Class** \| **Tree** \| **Deprecated** \| **Index** \| **Help** \| **Weka's home** | |  |
| **PREV CLASS**   **NEXT CLASS** | **FRAMES**    **NO FRAMES**     **All Classes** |  |
| SUMMARY: NESTED \| FIELD \| CONSTR \| METHOD | DETAIL: FIELD \| CONSTR \| METHOD |  |


---


## weka.core Class FindWithCapabilities

```
java.lang.Object
  weka.core.FindWithCapabilities
```

**All Implemented Interfaces:**: CapabilitiesHandler, OptionHandler, RevisionHandler

---

``` public class FindWithCapabilities extends java.lang.Object implements OptionHandler, CapabilitiesHandler, RevisionHandler ```

Locates all classes with certain capabilities. One should keep in mind,
that works only with the default capabilities of a scheme and doesn't
take dependencies into account. E.g., a meta-classifier that could have
a base classifier handling numeric classes, but by default uses one with
a nominal class, will never show up in a search for schemes that handle
numeric classes.


Valid options are:

```
 All class and attribute options can be prefixed with 'not',
 e.g., '-not-numeric-class'. This makes sure that the returned
 schemes 'cannot' handle numeric classes.
```

```
 -num-instances <num>
  The minimum number of instances (default 1).
```

```
 -unary-class
  Must handle unray classes.
```

```
 -binary-class
  Must handle binary classes.
```

```
 -nominal-class
  Must handle nominal classes.
```

```
 -numeric-class
  Must handle numeric classes.
```

```
 -string-class
  Must handle string classes.
```

```
 -date-class
  Must handle date classes.
```

```
 -relational-class
  Must handle relational classes.
```

```
 -missing-class-values
  Must handle missing class values.
```

```
 -no-class
  Doesn't need a class.
```

```
 -unary-atts
  Must handle unary attributes.
```

```
 -binary-atts
  Must handle binary attributes.
```

```
 -nominal-atts
  Must handle nominal attributes.
```

```
 -numeric-atts
  Must handle numeric attributes.
```

```
 -string-atts
  Must handle string attributes.
```

```
 -date-atts
  Must handle date attributes.
```

```
 -relational-atts
  Must handle relational attributes.
```

```
 -missing-att-values
  Must handle missing attribute values.
```

```
 -only-multiinstance
  Must handle multi-instance data.
```

```
 -W <classname>
  The Capabilities handler to base the handling on.
  The other parameters can be used to override the ones
  determined from the handler. Additional parameters for
  handler can be passed on after the '--'.
  Either '-W' or '-t' can be used.
```

```
 -t <file>
  The dataset to base the capabilities on.
  The other parameters can be used to override the ones
  determined from the handler.
  Either '-t' or '-W' can be used.
```

```
 -c <num>
  The index of the class attribute, -1 for none.
  'first' and 'last' are also valid.
  Only in conjunction with option '-t'.
```

```
 -superclass
  Superclass to look for in the packages.
```

```
 -packages
  Comma-separated list of packages to search in.
```

```
 -generic
  Retrieves the package list from the GenericPropertiesCreator
  for the given superclass. (overrides -packages <list>).
```

```
 -misses
  Also prints the classname that didn't match the criteria.
```

**Version:**
:   $Revision: 1.5 $

**Author:**
:   fracpete (fracpete at waikato dot ac dot nz)

**See Also:**: `Capabilities`, `Capabilities.Capability`, `GenericPropertiesCreator`

---

**Constructor Summary**

`FindWithCapabilities()`


| **Method Summary** | |
| --- | --- |
| `void` | `disable(Capabilities.Capability c)`             disables the given capability. |
| `void` | `disableNot(Capabilities.Capability c)`             disables the given "not to have" capability. |
| `void` | `enable(Capabilities.Capability c)`             enables the given capability. |
| `void` | `enableNot(Capabilities.Capability c)`             enables the given "not to have" capability. |
| `java.util.Vector` | `find()`             returns a list with all the classnames that fit the criteria. |
| `Capabilities` | `getCapabilities()`             The capabilities to search for. |
| `java.lang.String` | `getClassIndex()`             returns the current current class index, -1 if no class attribute. |
| `java.lang.String` | `getFilename()`             returns the current filename for the dataset to base the capabilities on. |
| `CapabilitiesHandler` | `getHandler()`             returns the current set CapabilitiesHandler to generate the dataset for, can be null. |
| `java.util.Vector` | `getMatches()`             returns the matches from the last find call. |
| `java.util.Vector` | `getMisses()`             returns the misses from the last find call. |
| `Capabilities` | `getNotCapabilities()`             The "not to have" capabilities to search for. |
| `java.lang.String[]` | `getOptions()`             Gets the current settings of this object. |
| `java.lang.String` | `getRevision()`             Returns the revision string. |
| `boolean` | `handles(Capabilities.Capability c)`             returns true if the given capability can be handled. |
| `boolean` | `isEnabled(Capabilities.Capability c)`             whether the given capability is enabled. |
| `boolean` | `isEnabledNot(Capabilities.Capability c)`             whether the given "not to have" capability is enabled. |
| `java.util.Enumeration` | `listOptions()`             Returns an enumeration describing the available options. |
| `static void` | `main(java.lang.String[] args)`             Executes the location of classes with parameters from the commandline. |
| `void` | `setCapabilities(Capabilities c)`             Uses the given Capabilities for the search. |
| `void` | `setClassIndex(java.lang.String value)`             sets the class index, -1 for none, first and last are also valid. |
| `void` | `setFilename(java.lang.String value)`             Sets the dataset filename to base the capabilities on. |
| `void` | `setHandler(CapabilitiesHandler value)`             sets the Capabilities handler to generate the data for. |
| `void` | `setNotCapabilities(Capabilities c)`             Uses the given "not to have" Capabilities for the search. |
| `void` | `setOptions(java.lang.String[] options)`             Parses a given list of options. |

**Methods inherited from class java.lang.Object**

`equals, getClass, hashCode, notify, notifyAll, toString, wait, wait, wait`

**Constructor Detail**

### FindWithCapabilities

```
public FindWithCapabilities()
```


**Method Detail**

### listOptions

```
public java.util.Enumeration listOptions()
```

:   Returns an enumeration describing the available options.

    :   **Specified by:**: `listOptions` in interface `OptionHandler`
    :   **Returns:**: an enumeration of all the available options.

---


### setOptions

```
public void setOptions(java.lang.String[] options)
                throws java.lang.Exception
```

:   Parses a given list of options.

    :   **Specified by:**: `setOptions` in interface `OptionHandler`
    :   **Parameters:**: `options` - the list of options as an array of strings **Throws:**: `java.lang.Exception` - if an option is not supported

---


### getOptions

```
public java.lang.String[] getOptions()
```

:   Gets the current settings of this object.

    :   **Specified by:**: `getOptions` in interface `OptionHandler`
    :   **Returns:**: an array of strings suitable for passing to setOptions

---


### setHandler

```
public void setHandler(CapabilitiesHandler value)
```

:   sets the Capabilities handler to generate the data for.

    :   **Parameters:**: `value` - the handler

---


### getHandler

```
public CapabilitiesHandler getHandler()
```

:   returns the current set CapabilitiesHandler to generate the dataset
    for, can be null.

    :   **Returns:**: the handler

---


### setFilename

```
public void setFilename(java.lang.String value)
```

:   Sets the dataset filename to base the capabilities on. It immediately
    loads the dataset and retrieves the capabilities from it.

    :   **Parameters:**: `value` - the filename of the dataset

---


### getFilename

```
public java.lang.String getFilename()
```

:   returns the current filename for the dataset to base the capabilities on.

    :   **Returns:**: the filename of the dataset

---


### setClassIndex

```
public void setClassIndex(java.lang.String value)
```

:   sets the class index, -1 for none, first and last are also valid.

    :   **Parameters:**: `value` - the class index

---


### getClassIndex

```
public java.lang.String getClassIndex()
```

:   returns the current current class index, -1 if no class attribute.

    :   **Returns:**: the class index

---


### enable

```
public void enable(Capabilities.Capability c)
```

:   enables the given capability.

    :   **Parameters:**: `c` - the capability to enable

---


### isEnabled

```
public boolean isEnabled(Capabilities.Capability c)
```

:   whether the given capability is enabled.

    :   **Parameters:**: `c` - the capability to enable **Returns:**: true if the capability is enabled

---


### disable

```
public void disable(Capabilities.Capability c)
```

:   disables the given capability.

    :   **Parameters:**: `c` - the capability to disable

---


### enableNot

```
public void enableNot(Capabilities.Capability c)
```

:   enables the given "not to have" capability.

    :   **Parameters:**: `c` - the capability to enable

---


### isEnabledNot

```
public boolean isEnabledNot(Capabilities.Capability c)
```

:   whether the given "not to have" capability is enabled.

    :   **Parameters:**: `c` - the capability to enable **Returns:**: true if the capability is enabled

---


### disableNot

```
public void disableNot(Capabilities.Capability c)
```

:   disables the given "not to have" capability.

    :   **Parameters:**: `c` - the capability to disable

---


### handles

```
public boolean handles(Capabilities.Capability c)
```

:   returns true if the given capability can be handled.

    :   **Parameters:**: `c` - the capability to check **Returns:**: true if the capability can be handled

---


### getCapabilities

```
public Capabilities getCapabilities()
```

:   The capabilities to search for.

    :   **Specified by:**: `getCapabilities` in interface `CapabilitiesHandler`
    :   **Returns:**: the capabilities to search for **See Also:**: `Capabilities`

---


### setCapabilities

```
public void setCapabilities(Capabilities c)
```

:   Uses the given Capabilities for the search.

    :   **Parameters:**: `c` - the capabilities to use for the search

---


### getNotCapabilities

```
public Capabilities getNotCapabilities()
```

:   The "not to have" capabilities to search for.

    :   **Returns:**: the capabilities to search for **See Also:**: `Capabilities`

---


### setNotCapabilities

```
public void setNotCapabilities(Capabilities c)
```

:   Uses the given "not to have" Capabilities for the search.

    :   **Parameters:**: `c` - the capabilities to use for the search

---


### getMatches

```
public java.util.Vector getMatches()
```

:   returns the matches from the last find call.

    :   **Returns:**: the matching classname from the last find run

---


### getMisses

```
public java.util.Vector getMisses()
```

:   returns the misses from the last find call.

    :   **Returns:**: the classnames that didn't match from the last find run

---


### find

```
public java.util.Vector find()
```

:   returns a list with all the classnames that fit the criteria.

    :   **Returns:**: contains all classnames that fit the criteria

---


### getRevision

```
public java.lang.String getRevision()
```

:   Returns the revision string.

    :   **Specified by:**: `getRevision` in interface `RevisionHandler`
    :   **Returns:**: the revision

---


### main

```
public static void main(java.lang.String[] args)
```

:   Executes the location of classes with parameters from the commandline.

    :   **Parameters:**: `args` - the commandline parameters


---


|  |  |  |
| --- | --- | --- |
| **Overview** \| **Package** \| **Class** \| **Tree** \| **Deprecated** \| **Index** \| **Help** \| **Weka's home** | |  |
| **PREV CLASS**   **NEXT CLASS** | **FRAMES**    **NO FRAMES**     **All Classes** |  |
| SUMMARY: NESTED \| FIELD \| CONSTR \| METHOD | DETAIL: FIELD \| CONSTR \| METHOD |  |


---
